# Insecticidal activity of garlic essential oil and their constituents against the mealworm beetle, *Tenebrio molitor* Linnaeus (Coleoptera: Tenebrionidae)

**DOI:** 10.1038/srep46406

**Published:** 2017-04-20

**Authors:** Angelica Plata-Rueda, Luis Carlos Martínez, Marcelo Henrique Dos Santos, Flávio Lemes Fernandes, Carlos Frederico Wilcken, Marcus Alvarenga Soares, José Eduardo Serrão, José Cola Zanuncio

**Affiliations:** 1 Instituto de Ciências Agrárias, Universidade Federal de Viçosa, 38810-000, Rio Paranaiba, Minas Gerais, Brasil; 2 Departamento de Entomologia, Universidade Federal de Viçosa, 36570-000, Viçosa, Minas Gerais, Brasil; 3 Departamento de Química, Universidade Federal de Viçosa, 36570-000, Viçosa, Minas Gerais, Brasil; 4 Departamento de Proteção de Plantas, Escola de Ciências Agronômicas, Universidade Estadual Paulista, 18603-970, Botucatu, Brasil; 5 Departamento de Agronomia, Universidade Federal dos Vales do Jequitinhonha e Mucuri, 391000-000 Diamantina, Minas Gerais, Brasil; 6 Departamento de Biologia Geral, Universidade Federal de Viçosa, 36570-000, Viçosa, Minas Gerais, Brasil.

## Abstract

This study evaluated the insecticidal activity of garlic, *Allium sativum* Linnaeus (Amaryllidaceae) essential oil and their principal constituents on *Tenebrio molitor*. Garlic essential oil, diallyl disulfide, and diallyl sulfide oil were used to compare the lethal and repellent effects on larvae, pupae and adults of *T. molitor*. Six concentrations of garlic essential oil and their principal constituents were topically applied onto larvae, pupae and adults of this insect. Repellent effect and respiration rate of each constituent was evaluated. The chemical composition of garlic essential oil was also determined and primary compounds were dimethyl trisulfide (19.86%), diallyl disulfide (18.62%), diallyl sulfide (12.67%), diallyl tetrasulfide (11.34%), and 3-vinyl-[4H]-1,2-dithiin (10.11%). Garlic essential oil was toxic to *T. molitor* larva, followed by pupa and adult. In toxic compounds, diallyl disulfide was the most toxic than diallyl sulfide for pupa > larva > adult respectively and showing lethal effects at different time points. Garlic essential oil, diallyl disulfide and diallyl sulfide induced symptoms of intoxication and necrosis in larva, pupa, and adult of *T. molitor* between 20–40 h after exposure. Garlic essential oil and their compounds caused lethal and sublethal effects on *T. molitor* and, therefore, have the potential for pest control.

The mealworm beetle, *Tenebrio molitor* Linnaeus (Coleoptera: Tenebrionidae) is a pest of stored products such as starches, food for cats and dogs, and pasta. This insect may also infest broken grains of *Zea mays* (L.) (Poales: Poaceae), *Triticum aestivum* (L.) (Poales: Poaceae) and *Glycine max* (L.) (Fabales: Fabaceae)^1^,^2^,^3^. The presence of *T. molitor* in stored grain and bran can contaminate food with fragments of the body, faeces and indirectly by saprophytic microorganisms causing loss of food quality^4^,^5^,^6^. *T. molitor* causes losses up to 15% of grains and flour production in worldwide^7^,^8^,^9^.

*T. molitor* is controlled primarily with chemical insecticides, but this method has restrictions against stored product insects^10^, due to residual toxicity and insect resistance^11^, especially in countries with extensive cereal production for export and domestic consumption^10^,^12^. Chemical control of this insect can be achieved by methyl bromide and phosphine treatment; however, fumigants cannot kill the eggs of storage pests and several issues have been discussed in the employment of insecticides, such as residue, environment impact and toxicity to humans^10^,^12^. Economic, social and environmental concerns have caused a gradual change to reduce chemical control in starches and stored products^11^,^12^,^13^. More selective and biodegradable products, including “green pesticides”, can reduce the use of synthetic chemicals in warehouses^14^,^15^.

Plant essential oils have favorable ecotoxicological properties (low toxicity to humans, further degradation, and lower environmental impact), making them suitable to managing insects in organic farming^16^,^17^. These oils are plants secondary metabolites and include alkaloids, amides, chalcones, flavones, kawapirones, lignans, neolignans or phenols which are important in insect-plant relationships^14^,^15^,^18^. In this sense, essential oils represent an alternative for pest control as repellents, deterrent of oviposition and feeding, growth regulators, and toxicity to insects with low pollution and quick degradation in the environmental^16^,^17^. Various studies have focused on the possibility of using plant essential oils for application to stored grain to control insect pests^19^,^20^,^21^.

Garlic, *Allium sativum* Linnaeus (Amaryllidaceae), is a native of temperate western Asia and has been used throughout the world as a food spice and medicine^22^. Antimicrobial^23^, cardiovascular^24^, anticancer^25^, hypo- and hyper-glycaemic, and other beneficial properties of garlic have been reported^26^. In different studies, garlic essential oil was demonstrated to possess insecticidal activity against *Blattella germanica* Linnaeus (Blattodea: Blatellidae)^27^, *Lycoriella ingénue* Dufour (Diptera: Sciaridae)^28^, *Reticulitermes speratus* Kolbe (Isoptera: Rhinotermitidae)^29^, and several grain storage insects as *Ephestia kuehniella* Zeller (Lepidoptera: Pyralidae), *Sitophilus oryzae* Linnaeus, *Sitophilus zeamais* Motschulsky (Coleoptera: Curculionidae), and *Tribolium castaneum* Herbst (Coleoptera: Curculionidae)^30^,^31^. There are a variety of insecticides that have toxicological properties, deterrents, and repellents used for the control of *T. molitor*; however, essential oil of garlic could be an alternative for the control in stored products. Identification of toxic compounds of garlic is important in understanding toxicity as it relates to pest control. In this study, we hypothesized that garlic essential oil and their constituents have insecticidal activity in *T. molitor*.

We examined the insecticidal activity of garlic essential oil as well as compounds identified on *T. molitor*, explain in various experiments: (i) garlic oil composition, (ii) toxicity test, (iii) lethal time test, (iv) repellency index and, (v) respiration rate, in order to contribute for the development of new strategies for controlling this insect pest affecting an important source of food.

## Results

### Susceptibility of *T. molitor* exposed to garlic essential oil

Mortality of *T. molitor* was obtained with 16 and 32% (w/v) of the garlic essential oil and three different lethal concentration levels (LC_50_ and LC_90_) were estimated by Probit (*X*^*2*^, P < 0.0001) (Table 1). The LC_50_ and LC_90_ values indicated that garlic essential oil was the most toxic to *T. molitor* larvae (*X*^*2*^ = 260.1, df = 5), followed by pupae (*Χ*^*2*^ = 149.5, df = 5) and adults (*Χ*^*2*^ = 46.6, df = 5). Mortality was always <1% in the control.

### Garlic essential oil composition

**Figure 1:**
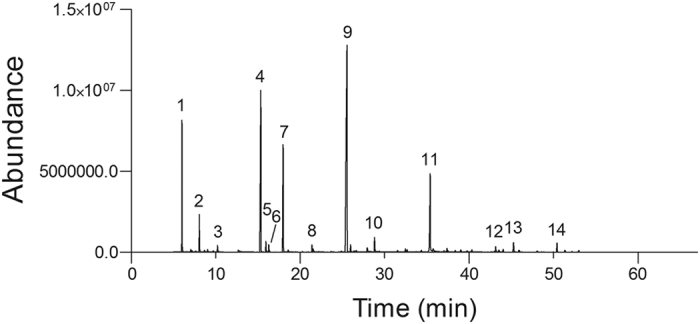
Gas chromatogram profiles of peak retention of compounds of the garlic essential oil: Diallyl sulfide (1), methyl allyl disulfide (2), dimethyl trisulfide (3), diallyl disulfide (4), diallyl tetrasulfide (5), unknown (6), methyl allyl trisulfide (7), 3-vinyl-[4H]-1,2-dithiin (8), allyl trisulfide (9), unknown (10), 1,4-dimethyl tetrasulfide (11), diallyl trisulfide (12), unknown (13), and unknown (14).

A total of 14 compounds from the garlic essential oil were obtained, 10 compounds were identified and 4 unknown which accounted for 97.54% of the total composition (Fig. 1, Table 2, Supplementary Fig. 1). The primary compounds of the garlic essential oil were dimethyl trisulfide (19.86%), diallyl disulfide (18.62%), diallyl sulfide (12.67%), diallyl tetrasulfide (11.34%), and 3-vinyl-[4 H]-1,2-dithiin (10.11%), followed by diallyl trisulfide (5.74%), allyl trisulfide (4.41%), 1,4-dimethyl tetrasulfide (4.06%), allyl disulfide (3.95%), methyl allyl disulfide (3.87%), and methyl allyl trisulfide (3.76%).

### Toxicity assessment of compounds

The toxicity of commercially obtained diallyl sulfide and diallyl disulfide in *T. molitor* were estimated by Probit (*X*^*2*^, P < 0.0001) and evaluated at different concentrations (Table 3). Toxicity was higher with diallyl disulfide, while diallyl sulfide was lower. Dose-response bioassays showed optimal results with diallyl disulfide by pupae (*Χ*^*2*^ = 46.45, df = 5) with a LC_50_ = 55.13 mg mL^−1^ and LC_90_ = 109.1 mg mL^−1^, followed larvae (*Χ*^*2*^ = 76.64, df = 5) with a LC_50_ = 57.68 mg mL^−1^ and LC_90_ = 154.3 mg mL^−1^, and adults (*Χ*^*2*^ = 31.32, df = 5) with a LC_50_ = 81.52 mg mL^−1^ and LC_90_ = 168.1 mg mL^−1^. The LC_50_ and LC_90_ values indicated that diallyl sulfide was toxic to pupae (*X*^*2*^ = 67.68, df = 5) with a LC_50_ = 48.86 mg mL^−1^ and LC_90_ = 210.5 mg mL^−1^, followed by larvae (*Χ*^*2*^ = 7.43, df = 5) with a LC_50_ = 117.1 mg mL^−1^ and LC_90_ = 222.8 mg mL^−1^, and adults (*Χ*^*2*^ = 50.83, df = 5) with a LC_50_ = 85.97 mg mL^−1^ and LC_90_ = 222.8 mg mL^−1^. Mortality was always <1% in the control.

### Lethal time of toxic compounds and garlic essential oil in *T. molitor*

**Figure 2:**
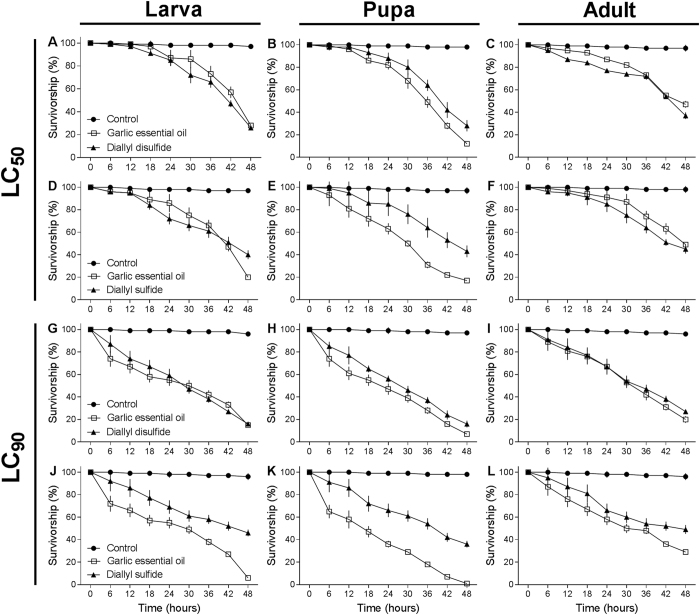
Survivorship of *Tenebrio molitor* after 48 h topical applied with a LC_50_ and LC_90_ garlic essential oil *vs* toxic compounds: larva (**A**,**D**,**G**,**J**), pupa (**B**,**E**,**H**,**K**), and adult (**C**,**F**,**I**,**L**) (control insects were applied with water). Control (⦁), garlic essential oil (◻), diallyl disulfide and diallyl sulfide (▴).

Larvae, pupae, and adults of *T. molitor* applied with LC_50_ and LC_90_ concentrations of garlic essential oil *vs* toxic compounds showed lethal effects at different time points (Fig. 2). However, LT_50_ values from topical application assays showed that diallyl sulfide took longer to kill insects than the diallyl disulfide and garlic essential oil. At a high LC_50_ concentration, diallyl sulfide took longer to kill the larvae (t = 1.29, P < 0.001), pupae (t = 4.23, P < 0.001), and adults (t = 2.16, P < 0.001) with LT_50_ values of 45.9 ± 0.52 h, 40.1 ± 0.38 h, and 54.2 ± 0.85 h, respectively. Diallyl disulfide took less time to kill the larvae (t = 3.24, P < 0.001), pupae (t = 4.16, P < 0.001), and adults (t = 2.12, P < 0.001) with LT_50_ values of 40.7 ± 0.14 h, 43.9 ± 0.52 h, and 49.7 ± 0.91 h, respectively. At a high LC_90_ concentration, diallyl sulfide took longer to kill the larvae (t = 4.45, P < 0.001), pupae (t = 4.51, P < 0.001), and adults (t = 3.96, P < 0.001) with LT_50_ values of 36.8 ± 0.85 h, 33.3 ± 0.43 h, and 40.3 ± 0.27 h, respectively. Diallyl disulfide took less time to kill the larvae (t = 4.31, P < 0.001), pupae (t = 4.31, P < 0.001), and adults (t = 4.08, P < 0.001) with LT_50_ values of 20.3 ± 0.45 h, 21.4 ± 0.16 h, and 27.7 ± 0.75 h, respectively.

**Figure 3:**
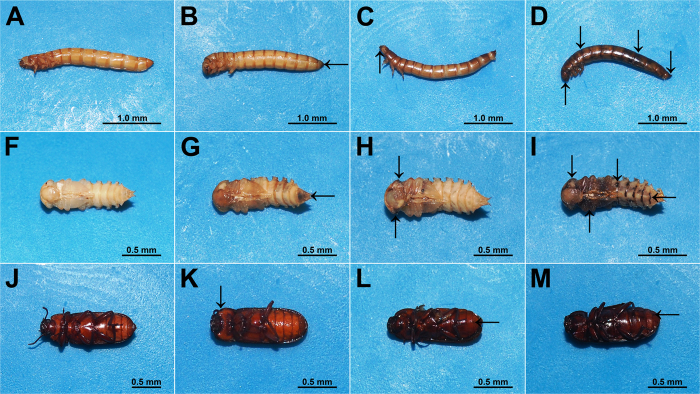
Time-course of garlic essential oil and toxic compounds on larva, pupa and adult of *Tenebrio molitor* after application to level LC_90_. Control (**A**,**E**,**I**) and sequential necrosis effects at 12 h (**B**,**F**,**J**), 24 h (**C**,**G**,**K**), and 48 h (**D**,**H**,**L**). Necrosis point (arrows).

The CL_50_ and CL_90_ values of garlic essential oil, diallyl disulfide and diallyl sulfide induced symptoms of intoxication in larvae and adults of *T. molitor*, such as progressive paralysis, reduced food consumption, and regurgitation. Necrosis was observed in larvae, pupae and adults on the area applied, mainly in mouthparts, pronotum, legs, abdomen segments, and anus (Fig. 3).

### Repellency test

**Figure 4:**
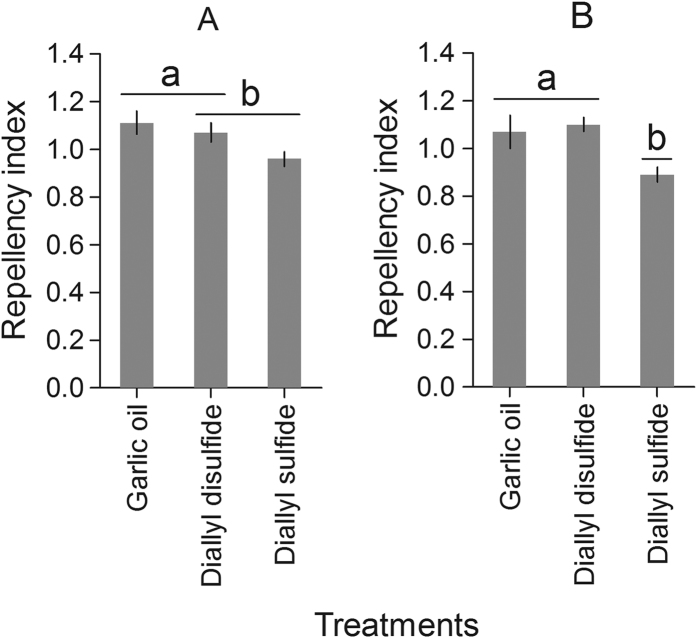
Repellency of *Tenebrio molitor* by the garlic essential oils and toxic compounds to level LC_90_ application on larvae (**A**) and adult (**B**). Treatments (Means ± SD) differ at P < 0.05 (Tukey’s mean separation test).

The larvae of *T. molitor* repellency index by garlic essential oil and compounds with concentrations estimated for the LC_90_ values differ between them (F_1,17_ = 7.61, P < 0.05) (Fig. 4A). The essential oil of garlic was the most repellent (RI = 1.11 ± 0.05), followed by that of the diallyl disulfide (RI = 1.07 ± 0.04), and diallyl sulfide (RI = 0.96 ± 0.03). The repellency index for adults of *T. molitor* differed with the concentration of the garlic essential oil and compounds, the estimated LC_90_ values (F_1,17_ = 6.81, P < 0.05) (Fig. 4B). Diallyl disulfide (RI = 1.11 ± 0.03) and garlic essential oil (RI = 1.07 ± 0.07) were the most repellent followed by diallyl sulfide (RI = 0.89 ± 0.03).

### Respiration rate

**Figure 5:**
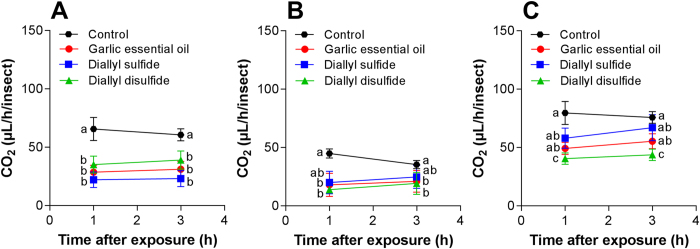
Respiration rate (Mean ± SE) of *Tenebrio molitor* after exposure to garlic essential oils and toxic compounds to level LC_90_ application on larvae (**A**), pupa (**B**), and adult (**C**). Treatments (Means ± SD) differ at P < 0.05 (Tukey’s mean separation test).

The respiration rate (μL of CO_2_ h^−1^/insect) of *T. molitor* was significantly different for garlic essential oil and compounds with concentrations estimated for the LC_90_ values in larva (F_3,71_ = 6.84, P < 0.001), pupa (F_3,71_ = 44.35, P < 0.001), and adult (F_3,71_ = 10.45, P < 0.001). Respiration rate observed between 1 and 3 h were different in larva (F_2,71_ = 4.95, P < 0.001), pupa (F_2,71_ = 12.75, P < 0.001), and adult (F_2,71_ = 58.23, P < 0.001). The interaction treatments and time in was different in larva (F_3,71_ = 10.45, P < 0.001), but was not different in pupa (F_3,71_ = 0.15, P = 0.932) and adult (F_3,71_ = 3.19, P = 0.041). In general, garlic essential oil, diallyl disulfide, and diallyl sulfide reduced the respiration rate of *T. molitor* at 1 and 3 h after exposure (Fig. 5).

## Discussion

Insecticidal activity of the garlic essential oil and its compounds against the mealworm beetle, *T. molitor* were determined from the bioassays in the laboratory conditions. Garlic essential oil caused substantial mortality and repellency in larva, pupa, and adult stages. The best results were obtained with concentrations of 16 and 32% in *T. molitor* as reported for other stored grain pests according to the concentration of these products^11^,^32^. The susceptibility of stored pest products such as *S. oryzae, S. zeamais, Sitotroga cerealella* Oliver (Lepidoptera: Gelechiidae), and *T. castaneum* may vary with the exposure at garlic essential oil applied to the body of these insects or by fumigation^30^,^33^,^34^.

Different concentrations of the garlic essential oil showed toxic effects on larva, pupa and adult of *T. molitor* 48 h after topical application. The dose-response bioassay confirmed toxicity against *T. molitor*, reaching a 90% mortality rate. Increasing concentrations of garlic essential oil in this insect have shown immediate toxic responses within 12 h of application^29^,^30^,^35^,^36^. Comparing the contact toxicity of garlic essential oil on developmental stages of *T. molitor*, the larva was significantly more susceptible followed by pupa and adult. The LC_50_ and LC_90_ of larva (0.77–1.36%), pupa (2.37–4.01%), and adult (2.03–4.73%) indicate that small quantities of the garlic essential oil are toxic in this insect, being more tolerant with age.

The chemical composition of the garlic essential oil revealed 14 compounds detected, 10 identified and quantified in terms of relative percentages. In particular, diallyl sulfide, diallyl disulfide, diallyl tetrasulfide, dimethyl trisulfide, and 3-vinyl-[4H]-1,2-dithiin were the main compounds that were detected in garlic essential oil. The result is in accordance with those of previous reports^30^,^36^,^37^. The above compounds are produced as a result of the degradation of allicin under the harsh thermal treatment in the hydrodistillation procedure. Allicin is not a stable compound and readily degrades *via* several pathways to form the secondary products of various sulfides contributing the characteristic flavour and odour of garlic^38^,^39^. Various studies indicated that the rapid decomposition of allicin in garlic aqueous extract involved transformation mainly to diallyl sulfides^40^,^41^. In this study, the main compounds of garlic essential oil are sulfur compounds (thiosulfinates) such as diallyl sulfide, diallyl disulfide, and diallyl tetrasulfide. However, there are considerable variations in the chemical composition of garlic essential oil where 3-vinyl-[4H]-1,2-dithiin is a main compound. In general, the diallyl sulfide, diallyl disulfide, and diallyl tetrasulfide are the most abundant constituents of fresh garlic oil and commercially can be variations in the relative proportions of these compounds^38^,^39^,^40^,^41^.

Diallyl sulfide and diallyl disulfide demonstrated toxic activity on different developmental stages of *T. molitor*. Diallyl disulfide have stronger contact toxicity in larvae (LC_50_ = 57.68 mg mL^−1^), pupae (LC_50_ = 55.13 mg mL^−1^), and adult (LC_50_ = 81.52 mg mL^−1^), than diallyl sulfide in larvae (LC_50_ = 117.1 mg mL^−1^), pupae (LC_50_ = 48.86 mg mL^−1^), and adult (LC_50_ = 85.97 mg mL^−1^). Garlic essential oil and its constituents, diallyl sulfide and diallyl disulfide have been highly toxic to *S. zeamais* and *T. castaneum*^30^,^35^ at different developmental stages as well as other insects^29^,^36^. Our results showed that *T. molitor* was more susceptible in the pupal stage followed by larvae and adults exposed to diallyl sulfide and diallyl disulfide. One possible explanation for the developmental stages difference is that efficacy may be affected by the penetration of the garlic compounds into the body and the ability of the insect to metabolize these compounds.

The insects exposed to the garlic essential oil and toxic compounds displayed altered locomotion activity, and muscle contractions that were observed in detailed descriptions at high concentrations in LC_50_ and LC_90_ test. In some individuals, the paralysis was constant with concentrations near the LC_50_ without recovery signs. Paralysis and muscle contractions in individuals of *T. molitor* at LC_50_ can be explained by the toxic effect in the nervous system of the same. The rapid toxicity of essential oils and their constituents in insects indicates neurotoxic action as reported to *Delia radicum* Linnaeus, *Musca domestica* Linnaeus (Diptera: Muscidae), *Cacopsylla chinensis* (Yang et Li), and *Diaphorina citri* Kuwayama (Hemiptera: Psyllidae) with hyperactivity, hyperextension of the legs and abdomen and rapid knock-down effect or immobilization^42^,^43^,^44^. Acetylcholinesterase is an enzyme that has been shown to be inhibited by garlic compounds and can act only or in synergism as diallyl disulfide, diallyl trisulfide, and allicin^45^,^46^. The presence of the diallyl sulfide in garlic compounds may be responsible for the toxic effect in *T. molitor* and may cause inhibition by cross-linking with essential thiol compounds in enzyme structures, altering the functional shape of the protein and denaturalization^47^.

The toxic compounds of garlic essential oil induced mortality in larva, pupa and adult of *T. molitor* within a short period of time. The LT_50_ of *T. molitor* larva applied with LC_90_ diallyl disulfide and diallyl sulfide was approximately 20 and 36 h, pupae was 21 and 33 h, and adults was 27 and 40 h, respectively. Toxic compounds affect multiple regions of the insect body over a period of time, ranging from one to 20–40 h for death. In this period, the necrotic areas were increasing progressively on the insect body. The comparative effects on *T. molitor* between garlic essential oil and toxic compounds were observed at various time points. An essential oil of quick action should be preferred for protection of products stored to be able to prevent feeding and avoid or reduce damage by insect pests^11^,^15^,^16^.

The repellency test indicated that garlic essential oil and diallyl disulfide has a greater effect on *T. molitor* behavior than diallyl sulfide had little effect. Odor produced from volatile compounds was repulsive to larvae and adults of *T. molitor* and was observed during early hours to finished bioassay. Monosulfides, disulfides, and trisulfide breakdown products of the thiosulfinates are volatile compounds and toxic to insect herbivores^34^,^43^,^48^. These compounds are garlic majority compounds and toxic to pests of stored products such as *S. zeamais* and *T. castaneum*^35^. Garlic essential oil has high percentage of diallyl disulfide as main volatile compound and repellent properties to *S. zeamais* and *T. castaneum*^30^. Our results suggest that garlic essential oil, diallyl disulfide, and diallyl sulfide have high activities of behavioral deterrence against *T. molitor*, as evaluated by the behavioral responses of larvae and adults to different odor sources and the number of insects repelled, indicating their potential to the pest control in stored products.

The garlic essential oil and their toxic constituents compromised the respiration rate of *T. molitor* up to 3 h after exposure, likely reflecting in behavior response and subsequently, the locomotors activity. Respiratory rate and body mass of insects can represent the sum of the energy demands of the physiological processes of insects that are necessary to produce defense mechanisms against the essential oils and toxic compounds^49^,^50^,^51^. Thus, low respiration rate is an indicator of physiological stress, and essential oils can compromise insect respiration by impairing muscle activity, leading to paralysis^49^,^50^,^51^. Several studies of plant volatile and their constituents were shown to effectively disrupt the recognition process of the host substrate and influence the walking behavior on insects^52^,^53^. The absorption of a fumigant by an insect is positively correlated with its respiration rate^54^. In this study, adults of *T. molitor* have low respiration rate caused by garlic essential oil and their constituents, resulting in physiological costs due to energy reallocation from other basic physiological processes. This favors the use of these toxic compounds by fumigant action and can cause significant negative effects on *T. molitor*.

This study showed the potential of garlic essential oil and main compounds to control the *T. molitor* in starches and stored products. In order to prevent or retard the development of insecticide resistance, the toxicity and repellency effects of garlic essential oil and toxic compounds on *T. molitor* show that can be used individually or mixture to management populations. The lethalities of diallyl sulfide and diallyl disulfide on *T. molitor* may have advantages by their mode of action on this insect. These findings show that the compounds of garlic essential oil are a potential source of insecticidal compounds and warrants further exploration.

## Methods

### Insects

Individuals of *T. molitor* were obtained from the Laboratory of Biological Control of the Institute of Applied Biotechnology to Agriculture (BIOAGRO, Universidade Federal de Viçosa) in Viçosa, Minas Gerais State, Brasil. Adults of *T. molitor* were kept in plastic trays (60 cm long × 40 cm wide × 12 cm) and maintained at 25 ± 1 °C, 70 ± 10% RH and 12:12 h L:D photoperiod. These adults were fed *ad libitum* with wheat bran (12% protein, 2% lipids, 75% carbohydrates and 11% mineral/sugar), pieces of sugarcane, *Saccharum officinarum* (L.) (Poaceae) and chayote *Sechium edule* (Jacq.) Swartz (Cucurbitaceae). Sheets of paper were placed on the substrate to prevent the stress from insects and facilitate oviposition. Healthy larvae, pupae and adults of *T. molitor* without amputations, apparent malformations or nutritional deficiencies were used in the bioassays.

### Garlic essential oil

The essential oil of garlic, *A. sativum* used in this study was commercial sample from Ferquima Industry & Commerce Ltda. (Vargem Grande Paulista, São Paulo State, Brasil), produced in industrial scale by hydrodistillation drag of water vapor^55^. Ferquima Industry & Commerce Ltda is accredited by the Ministry of Agriculture, Livestock and Supply of Brazil and by international organizations according to ISO Guide^56^. This provides Brazilian producers with licenses, and ensures unrestricted access to major world organic product markets.

### Mortality test

Garlic essential oil efficacy was determined by calculating the lethal concentrations (LC_50_, and LC_90_) values under laboratory conditions and conducted in triplicate. Six concentrations of garlic essential oil besides the control (acetone) were adjusted in 1 mL of stock solution (essential oil and acetone): 1, 2, 4, 8, 16 and 32% (w/v). Aliquots were taken from the stock solution and mixed with acetone in 5 mL glass vials. Different concentrations of the garlic essential oil were applied in 1 uL solution on the thorax of larva, pupa and adult of *T. molitor*, using a micropipette. For each developmental stage, fifty insects were used per concentration and were placed individually in Petri dishes (Ø 90 mm × 15 mm) with an absorbent paper, fed with chayote and sugarcane *ad libitum* (larvae and adults) and maintained in the dark. The number of dead insects was in each cage was counted after essential oil exposure for 48 h after application. Rates were calculated with a correction for natural mortality^57^.

### Identification of garlic essential oil compounds

Quantitative analyses of the garlic essential oil formulation were performed using a gas chromatograph (GC-17A series instrument, Shimadzu, Kyoto, Japan) equipped with a flame ionization detector (FID). The following chromatographic conditions were used: a fused silica capillary column (30 m × 0.22 mm) with a DB-5 bonded phase (0.25 μm film thickness); carrier gas N_2_ at a flow rate of 1.8 mL min^−1^; injector temperature 220 °C; detector temperature 240 °C; column temperature programmed to begin at 40 °C (remaining isothermal for 2 min) and then to increase at 3 °C min^−1^ to 240 °C (remaining isothermal at 240 °C for 15 min); injection volume 1.0 μL (1%w/v in CH_2_Cl_2_); split ratio 1:10; column pressure 115 kPa.

The compounds were identified using a gas chromatograph coupled with a mass detector GC/MS (GCMS-QP 5050 A; Shimadzu, Kyoto, Japan). The injector and detector temperatures were 220 °C and 300 °C, respectively. The initial column temperature was 40 °C for 3 min, with a programmed temperature increase of 3 °C/min to 300 °C where it was maintained for 25 min. The split mode ratio was 1:10. One microliter of the garlic essential oil containing 1% (w/v in dichloromethane) was injected, helium was used as the carrier gas with a flow rate constant of 1.8 mL^−1^ on the Rtx^®^-5MS capillary column (30 m, 0.25 mm × 0.25 μm; Bellefonte, USA) using the Crossbond^®^ stationary phase (35% diphenyl-65% dimethyl polysiloxane). Mass spectrometer was programmed to detect masses in the range of 29–450 Da with 70 eV ionization energy. Compounds were identified by comparisons of the mass spectra with those available in the NIST08, NIST11 library and Wiley Spectroteca Data Base (7^th^ edition), and by the Retention indices.

### Toxicity of commercial compounds of garlic essential oil in *T. molitor*

Diallyl sulfide and diallyl disulfide identified as toxic compounds in garlic essential oil, were obtained commercially. Diallyl sulfide (purity 97.0%) and diallyl disulfide (purity 70%) were purchased from Across Organics (New Jersey, USA). Six different concentrations of the commercial compounds besides the control (acetone) were adjusted in 1 mL of stock solution (treatment and acetone) used to calculate the lethal LC_50_ and LC_90_ concentrations: 5, 10, 20, 40, 80, and 160 mg mL^−1^. For each treatment, aliquots were taken from the stock solution and mixed with acetone in 5 mL glass vials. Different concentrations of the each treatment were applied in 1 uL solution on the body of larva, pupa and adult of *T. molitor*. Individuals were placed in Petri dishes with wheat bran and sugarcane. A total of 50 larvae, 50 pupae, and 50 adults were used for each concentration and mortality was evaluated for 48 h after application to compared with toxicity garlic essential oil, followed by rate corrections according to the Abbott’ formule^57^.

### Lethal time of toxic compounds and garlic essential oil in *T. molitor*

Toxicity of garlic essential oil and commercially obtained diallyl sulfide and diallyl disulfide at the calculated LC_50_ and LC_90_ concentrations were compared. Distilled water was used as a control. The LC_50_ and LC_90_ of each compound were applied in larvae, pupae, and adults of *T. molitor*. Insects were individualized in Petri dishes with wheat bran and sugarcane. A total of 240 larvae, 240 pupae, and 240 adults were used for each treatment, with a total of four replicates. Mortality was recorded every 6 h for 48 h, and estimated lethal time values for 50% mortality (LT_50_) were compared.

### Repellency test

Four Petri dishes (12 × 1.5 cm) were used as an arena, connected to a central board with plastic tubes (diameter 2 cm) at an angle of 45°. The other dishes were distributed around them in equidistant distances and two plates were put together symmetrically opposed. Two hundred and forty individuals (120 larvae and 120 adults) of *T. molitor* were released in the central board and the control group received sugarcane and chayote. A total of 5 uL of the estimated LC_90_ lethal concentration of garlic essential oil and toxic compounds were applied on absorbent filter paper (2 × 2 cm) placed in two opposite plates used as treatment and two opposite ones with 5 uL of distilled water for absorbent filter paper represented the control. Four replicates per treatment and control were evaluated by the number of individuals per plate after 24 hours calculating the repellency index (IR): RI = *2* *G*/(*L* + *P*), where G is the percentage of insects in the treatment and P is the percentage of insects in control. Treatments were classified as neutral if the index was equal to one (1); repellent, higher than one (1), and; attractive lower than one (1).

### Respiration rate

Respirometry bioassays were conducted 3 h after the beetles were exposed or not exposed to garlic essential oil and their constituents, as previously detailed. The doses used corresponded to the recorded LC_50_ of each toxic compound and control consisted of insect treated with distilled water. Carbon dioxide (CO_2_) (μL of CO_2_ h^−1^/insect) production was measured with a respirometer of the type CO_2_ Analyzer TR_3_C (Sable System International, Las Vegas, USA), using methodology adapted from previous studies^49^. Respirometers (25 mL) were used, each one holding 3 adults of *T. molitor* with mixed sex and connected to a closed system. CO_2_ production was measured after the insects were acclimated in the chambers for a period of 12 h at a temperature of 27 ± 2 °C. To quantify the CO_2_ produced inside each chamber, compressed oxygen gas (99.99% pure) was passed through the chamber at a flow of 100 mL min^−1^ for a period of 2 min. This airflow forces all produced CO_2_ molecules to pass through an infrared reader coupled to the system, which makes a continuous measurement of the CO_2_ produced by the insects and held inside each chamber. After CO_2_ measurement, the insects were removed from the chambers and then weighed using an analytical balance (Sartorius BP 210D, Göttingen, Germany). Respiration rate values were not normalized by body mass because this method masks the individual effects of the variables^58^. Six replicates were used for each treatment.

### Statistics

Lethal concentration (LC_50_ and LC_90_) and their confidence limits for garlic essential oil and toxic compounds were determined by logistic regression in dose-response assays based on the concentration Probit-mortality^59^ using XLSTAT-PRO (v.7.5) program for Windows^60^. Student’s t test was used for pairwise comparisons regarding lethal time effects in *T. molitor* using SAS User software (v. 9.0) for Windows^61^. Repellency data larvae and adults of *T. molitor* were transformed using formula 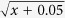 and analyzed by one-way ANOVA. A Tukey’s Honestly Significant test (HSD) was also used for comparisons of the means in the bioassays at 5% significance level using SAS User software. Respiration rate were subjected to two-way analyses of variance (time × insecticide treatment) and Tukey’s HSD test (P < 0.05) when appropriate (PROC GLM). As the time interval was assessed in different insect samples, they are not pseudoreplicates in time and therefore subject to regular two-way analyses of variance instead of repeated measures analyses of variance using SAS User software.

## Additional Information

**How to cite this article**: Plata-Rueda, A. *et al*. Insecticidal activity of garlic essential oil and their constituents against the mealworm beetle, *Tenebrio molitor* Linnaeus (Coleoptera: Tenebrionidae). *Sci. Rep.* **7**, 46406; doi: 10.1038/srep46406 (2017).

**Publisher's note:** Springer Nature remains neutral with regard to jurisdictional claims in published maps and institutional affiliations.

## Supplementary Material

Supplementary Information 1

## Figures and Tables

**Table 1:** Lethal concentrations of the garlic essential oil against different developmental stages of *Tenebrio molitor* after 48 hours exposure.

^1^IS	^2^LC	^3^VE	^4^IC	^5^*X*^*2*^
Larva	LC_50_	0.771	0.668–0.871	260.15
	LC_90_	1.365	1.236–1.544	
Pupa	LC_50_	2.371	2.171–2.615	149.51
	LC_90_	4.016	3.676–4.465	
Adult	LC_50_	2.032	1.751–2.326	46.65
	LC_90_	4.736	4.215–5.465	

^1^IS, insect stage; ^2^LC_50 and 99_, lethal concentration causing 50, 90 and 99% mortality; ^3^EV, estimated value; ^4^CI, confidential interval; ^5^*X*^*2*^, chi-squared value for the lethal concentrations and fiducial limits based on a log scale with significance level at P < 0.001.

**Table 2:** Chemical composition of the garlic essential oil.

Peak number	Compound	Formula	MM	RI	Ri	Rt	m/z
1	Diallyl sulfide	C6H10S	114	17.70	849	5.650	114.05
2	Methyl allyl disulfide	C4 H8 S2	120	4.60	911	7.850	122.00
3	Dimethyl trisulfide	C2H6S3	126	38.40	972	9.950	127.90
4	Diallyl disulfide	C6H10S2	146	5.40	1099	14.90	145.95
5	Diallyl tetrasulfide	C6H10S4	210	16.10	1601	15.56	147.95
6	Unknown	C6H10S2	146	14.9	1099	16.11	145.95
7	Methyl allyl trisulfide	C4 H8 S3	152	5.10	1128	17.42	110.90
8	3-vinyl-[4H]-1,2-dithiin	C6 H8 S2	144	26.40	1134	21.18	157.85
9	Allyl trisulfide	C6H10S3	178	4.90	1350	25.03	114.05
10	Unknown	C4 H8 S2	120	8.60	911	28.45	183.85
11	1,4-Dimethyl tetrasulfide	C6H10S4	210	6.60	1601	34.83	209.85
12	Diallyl trisulfide	C6H10S3	178	11.80	1350	43.26	113.05
13	Unknown	C6H10S3	178	5.40	1350	44.85	146.95
14	Unknown	C6H10S3	178	13.40	1350	49.91	186.95

MM - Molecular mass, RI - Relative intensity, Ri - Retention indices, Rt - Retention time, m/z - Molecular weight.

**Table 3:** Lethal concentrations of the diallyl sulfide and diallyl disulfide on different developmental stages of *Tenebrio molitor* after 48 hours exposure.

Compounds	^1^IS	^2^LC	^3^VE mg mL^−1^	^4^IC mg mL^−1^	^5^*X*^*2*^
Diallyl sulfide	Larva	LC_50_	117.1	104.9–132.4	7.43
		LC_90_	222.8	198.1–256.9	
	Pupa	LC_50_	48.86	36.71–61.85	67.68
		LC_90_	210.5	176.1–265.8	
	Adult	LC_50_	85.97	77.84–95.51	50.83
		LC_90_	247.5	207.3–311.7	
Diallyl disulfide	Larva	LC_50_	57.68	44.88–66.37	76.64
		LC_90_	154.3	136.9–177.8	
	Pupa	LC_50_	55.13	49.78–61.31	46.45
		LC_90_	109.1	98.77–122.3	
	Adult	LC_50_	81.52	57.96–91.25	31.32
		LC_90_	168.1	152.1–188.7	

^1^IS, insect stage; ^2^LC_50 and 99_, lethal concentration causing 50, 90 and 99% mortality; ^3^EV, estimated value; ^4^CI, confidential interval; ^5^*X*^*2*^, chi-squared value for the lethal concentrations and fiducial limits based on a log scale with significance level at P < 0.001.
