# Supplementary material for: Insecticidal activity of garlic essential oil and their constituents against the mealworm beetle, Tenebrio molitor Linnaeus (Coleoptera: Tenebrionidae)
Source: Sci Rep. 2017 Apr 20;7:46406. doi: 10.1038/srep46406 (PMC5397855; doi:10.1038/srep46406)
Supplement: Supplementary Information 1 [file srep46406-s1.doc]

**Insecticidal activity of garlic essential oil and their constituents against the mealworm beetle, *Tenebrio molitor* Linnaeus (Coleoptera: Tenebrionidae)**

Angelica Plata-Rueda^1^, Luis Carlos Martínez^2^, Marcelo Henrique Dos Santos^3^, Flávio Lemes Fernandes^1^, Carlos Frederico Wilcken^4^, Marcus Alvarenga Soares^5^, José Eduardo Serrão^6^, José Cola Zanuncio^2^

^1^Instituto de Ciências Agrárias, Universidade Federal de Viçosa, 38810-000, Viçosa, Minas Gerais, Brasil; angelicaplata@yahoo.com.mx; flaviofernandes@ufv.br

^2^Departamento de Entomologia, Universidade Federal de Viçosa, 36570-000, Viçosa, Minas Gerais, Brasil; lc.martinez@outlook.com; zanuncio@ufv.br

^3^Departamento de Química, Universidade Federal de Viçosa, 36570-000, Viçosa, Minas Gerais, Brasil; marceloh.santos@ufv.br

^4^Departamento de Proteção de Plantas, Escola de Ciências Agronômicas, Universidade Estadual Paulista, 18603-970, Botucatu, Brasil, e-mail: cwilken@fca.unesp.br

^5^Departamento de Agronomia, Universidade Federal dos Vales do Jequitinhonha e Mucuri, 391000-000 Diamantina, Minas Gerais, Brasil, e-mail: marcussoares@yahoo.com

^6^Departamento de Biologia Geral, Universidade Federal de Viçosa, 36570-000, Viçosa, Minas Gerais, Brasil; jeserrao@ufv.br

**Supplementary 1 (Fig. 1).** Chemical composition of garlic essential oil. Mass spectra of each compounds identified.

1. Diallyl sulfide

1. Methyl allyl disulfide

1. Dimethyl trisulfide

1. Diallyl disulfide

1. Diallyl tetrasulfide

1. C6H10S2

1. Methyl allyl trisulfide

1. 3-vinyl-[4H]-1,2-dithiin

1. Allyl trisulfide

1. C4 H8 S2

1. 1,4-dimethyl tetrasulfide

1. diallyl trisulfide

1. C6H10S3

1. C6H10S3
